# Supplementary material for: Implementing substance use services into acute care settings for pregnant and birthing people: A systematic scoping review of implementation and quality improvement strategies
Source: PLoS One. 2026 Mar 13;21(3):e0344389. doi: 10.1371/journal.pone.0344389 (PMC12987426; doi:10.1371/journal.pone.0344389)
Supplement: S2 File — (DOCX) [file pone.0344389.s002.docx]

**S2 File. Data extraction tool.**

| **#** | **Extraction Item** | **Responses** | |
| --- | --- | --- | --- |
| **Extraction Details** | | | |
| 1 | Extractor initials | Free text | |
| **Citation Details** | | | |
| 3 | Authors | Free text | |
| 4 | Publication year | Free text | |
| 5 | Title (copy and paste) | Free text | |
| 6 | Abstract (copy and paste) | Free text | |
| **Participants** | | | |
| 7 | Who is the actor of the strategy (the person/group enacting the strategy)?) [1] | Select all that apply:  Patients  Hospital providers  Hospital administrators  Educators  Community organization  States organizations (excluding PQCs)  Perinatal Quality Collaborative (PQCs)  Federal organization  Other (please specify): | |
| 8 | Who is the target of the actions? (i.e., the action target) [1] | Select all that apply:  Patients  Hospital providers  Hospital administrators  Community  States  Students (Medical/Nursing)  Other (please specify): | |
| 9 | What class of substance use does the strategy address? | Select all that apply:  Substance use disorder, generally  Opioids  Alcohol  Stimulants  Cannabis  Hallucinogens  Inhalants  Sedatives  Hypnotics/anxiolytics  Other (please specify): | |
| **Concept** | | | |
| 10 | Study type | Select one:  Program descriptives (only a description; no outcomes measured)  Quality improvement project  Observational (excluding quality improvement projects)  Experimental/Quasi-experimental/Pragmatic | |
| 11 | Methods | Select one:  Qualitative  Quantitative  Both  N/A (i.e., descriptive study without outcomes) | |
| 12 | Design | Select one:  Cross-sectional  Prospective  Retrospective | |
| 13 | Theories/Models/Frameworks guiding study design | Free text | |
| **Context** | | | |
| 14 | State, county, and/or city |  | |
| 15 | Region | Select all that apply:  Rural  Urban  Suburban  Other:  Not specified | |
| 16 | Target hospital setting of care delivery | Select all that apply:  Emergency Department  Inpatient  Labor/Delivery/NICU (specifically)  Other: | |
| 17 | Study period  (i.e., period of data collection or implementation) | Free text | |
| **Identify and classify strategies** | | | |
| 18 | What is the gap/problem/practice guideline that the strategy is addressing? (i.e., provider bias; naloxone distribution) | Free text | |
| 19 | What strategies/approaches were used? | 1. Strategy Type (using Leeman et al. [2])   1) Dissemination strategy  2) Implementation process strategy  3) Integration strategy  4) Capacity-building strategy  5) Scale-up strategy  Briefly describe the strategy (i.e., the actions, steps, or processes that needed to be enacted [1] (free text):  What is the dose of the strategy (i.e., duration, frequency) (free text): | |
| **Racial Equity** | | | |
| 20 | Does the study mention racial and/or ethnic equity? | 1. Yes/No 2. If yes, Is it explicit or implicit? (free text):   **explicit:** describes or names “racial equity”, “structural racism”, “structural competency”  **implicit:** acknowledges that racialized disparities exist, but not using equity-specific language   1. Copy/paste any specific definitions: 2. Where was it mentioned? (select all that apply):   Background/Introduction  Methods  Results  Discussion/Limitations  Other (please specify):   1. Was it operationalized in the study? Yes/No 2. Briefly describe (i.e., considered in study design, noted in discussion as future direction) (free text) | |
| **Outcomes** | | | |
| 21 | Are implementation outcomes measured? (As defined by Proctor et al.[3]) | 1. Yes/No 2. If yes, which outcomes?   Acceptability  Appropriateness  Feasibility  Adoption  Fidelity  Cost  Penetration  Sustainability  Other/Not listed (please specify):   1. At what level were the outcomes measured?   Community  Hospital  Providers  Patients   1. What were the results? (copy and paste from the results section): | |
| 22 | Are other clinical/service/process outcomes measured?  (i.e., overdose, acute care utilization) | 1. Yes/No 2. If yes, describe (copy and paste from methods): 3. What were the results (copy and paste from results)? 4. Any additional results of note? | |
| **Barriers or facilitators** | | | |
| 23 | Any reported barriers? (specific to this study, reported in results or discussion sections) | | Free text |
| 24 | Any reported facilitators? (specific to this study, reported in results or discussion sections) | | Free text |
| **Other notes** | | | |
| 25 | Any additional notes | | Free text |
| 26 | Exemplar quotes (qualitative or mixed methods studies only) | | Free text |

1. Proctor EK, Powell BJ, McMillen JC. Implementation strategies: recommendations for specifying and reporting. Implement Sci. 2013;8:139.

2. Leeman J, Birken SA, Powell BJ, Rohweder C, Shea CM. Beyond "implementation strategies": classifying the full range of strategies used in implementation science and practice. Implement Sci. 2017;12(1):125.

3. Proctor E, Silmere H, Raghavan R, Hovmand P, Aarons G, Bunger A, et al. Outcomes for implementation research: conceptual distinctions, measurement challenges, and research agenda. Adm Policy Ment Health. 2011;38(2):65-76.
