# Supplementary material for: Implementing substance use services into acute care settings for pregnant and birthing people: A systematic scoping review of implementation and quality improvement strategies
Source: PLoS One. 2026 Mar 13;21(3):e0344389. doi: 10.1371/journal.pone.0344389 (PMC12987426; doi:10.1371/journal.pone.0344389)
Supplement: S3 File — (DOCX) [file pone.0344389.s003.docx]

**S3 File. Detailed summary of studies from 2016-2023 that used implementation/QI strategies to integrate SUD services into acute care settings for pregnant or birthing people.**

| **Author, Year** | **Hospital setting** | **Implementer** | **Target population^a^** | **Target population sample size** | **Strategy description** | **Reported implementation outcomes^b^** | **Racial equity in design/**  **implementation** |
| --- | --- | --- | --- | --- | --- | --- | --- |
| 1. **Education and learning collaboratives** | | | | | | | |
| Ford et al., 2021[1] | Labor/  Delivery/ NICU | State PQC | Hospital providers | 1496 at baseline, 1684 at midpoint, 1898 final assessment | 32-month multi-site education collaborative that included education of SUD treatments, community/parent outreach and partnerships with local SUD treatment programs | Increased attitude scores  from baseline to midpoint (18.99 to 19.94, p< 0.0001) and maintained at final assessment | No |
| Hostetter et al., 2022[2] | Multiple | Educators | Students | 20 | 10-month experiential learning curriculum for third-year medical providing exposure to SUD and SUD treatments during pregnancy | Students rated the program highly feasible and acceptable; resources were useful, and the curriculum was a “valuable learning experience” | No |
| Laks et al., 2023[3] | Multiple | Educators | Hospital providers | - | Two-year Maternal Health Addiction fellowship in perinatal care and addiction medicine | The program was feasible and valuable, and led to improvements in clinical training/education, and collaboration between addiction medicine and OB/GYN | Yes |
| Merritt et al., 2022[4] | Multiple | Not specified | Hospital providers | 89 at time 1, 76 at time 2, 38 at time 3 | One-time educational session at a conference for healthcare professionals about substance use and caring for pregnant people with SUDs | Provider attitudes improved post-intervention; changes not sustained after 60-days  Adapted attitudes of health care providers survey mean (SD) scores: time 1= 38.24 (8.93), time 2= 32.71 (7.77), p<0.05; time 3=  37.08 (8.45), p<0.05] | No |
| Moore et al., 2023[5] | Multiple | Hospital providers, Educators | Hospital providers | 290 | 10-session ECHO program (30-minute didactic lecture followed by a 30-minute collaborative case review) for rural perinatal providers | The program was “reachable and well-attended”; Participants reported increased knowledge/awareness of SUD treatments and referral resources | No |
| Rudolf et al, 2018[6] | Multiple | Not specified | Hospital providers | 91 pre- and post-education | 120-minutes educational session on substance use in pregnancy, stigma, medication, and compassionate care | 13% decrease in stigma scores (p<0.001); 14% increase in compassion scores (p<0.002), 31% increase in knowledge scores (p<0.001), 24% increase in comfort level (p<0.001) and 17% increase in attitudes scores (p<0.001) | No |
| Shuman et al., 2022[7] | Multiple | Educators | Students | 11 | Arts-based intervention pilot that used creative writing and art to improve provider attitudes and reduce stigma towards perinatal patients | Improved attitudes toward perinatal substance use (t= 4.11, p=.002); participants were highly satisfied (mean=4.6/5, SD=0.1) | No |
| Stephenson et al., 2022[8] | Multiple | State PQC | Hospital providers | 369 | Development of a Statewide Speakers Bureau to disseminate best practices for OUD in pregnancy to local healthcare providers | 57.5% of providers were very confident that the education will improve their care of pregnant and postpartum people;  71% reported being very likely to apply the information to their practice | Yes |
| Tobin et al., 2018[9] | Labor/  Delivery/ NICU | Educators | Hospital providers | 206 | Two-hour seminar on evidence-based care with role playing | Knowledge of addiction, treatment, and neonatal withdrawal increased on average from an 87.3 pretest score to a 96.1 post-test score (p<0.001)  Most participants (96%) were able to correctly identify 3 clinical skills they would use with mothers with SUD | No |
| Walsh et al., 2017[10] | Multiple | Hospital providers | Hospital providers | 88 pre- and 78 post-education | 90-minute education session on withdrawal, treatment and compassionate care for SUD | Decreased stigma scores (p<0.015)  Increased compassion (p<0.036), knowledge (p<0.001), and attitudes (p<0.006) scores | No |
| Wands et al., 2022[11] | Multiple | Educators | Students | 85 | 20-minute simulation and debrief with a standardized patient portraying a pregnant person with SUD | Successfully demonstrated motivational interviewing skills and caring behaviors during the simulation | No |
| 1. **Clinical workflows and pathways** | | | | | | | |
| Kilaru et al., 2020[12] | Multiple | State agencies | Hospitals | 155 | State funded hospital incentive program where hospitals earned payments for designing clinical pathways for OUD | 76% of hospitals adopted  a pathway for post-discharge referrals and treatment for pregnant people with OUD | No |
| Lilly et al, 2019[13] | Multiple | Federal and state and agencies, State PQC, Hospital providers | Hospital administrators and providers | - | Collaborative workflow to screen and refer patients to outpatient SUD clinics from labor/delivery and ED | Program reached high-risk groups (including those in labor/delivery) | No |
| Naliboff et al., 2023[14] | Labor/  Delivery/ NICU | State PQC, Hospital providers, Community organization | Hospital providers, post-partum people | 17 providers, 197 post-partum people | Developed a workflow for universal distribution of a first aid kit with naloxone | 14 (82%) providers completed educational modules  192 (97%) postpartum people received overdose education and 186 (94%) accepted first aid kits, 150 (76%) accepted naloxone | No |
| Paterno et al., 2019[15] | Labor/  Delivery/ NICU | Hospital providers, Community organizations | Hospital providers | - | Nurse-led community-based pilot program to improve SUD screening and referral pathways at a maternal care practice and community hospital | 89.5% of pregnant people (17/19) received at least one prenatal referral to a community resource post-intervention, compared with 57.9% (11/19) pre-intervention | No |
| Snyder et al., 2021[16] | Emergency | Federal and state agencies, Hospital administrators and providers | Hospitals | 52 | Large-scale implementation project with technical assistance to improve uptake of low-threshold ED buprenorphine | 92.3% of hospitals initiated buprenorphine for pregnant people | No |
| Stone et al., 2023[17] | Labor/  Delivery/ NICU | Hospital providers | Hospital providers | - | Electronic health record order set for naloxone distribution to postpartum patients at an increased risk of opioid overdose | Postpartum patients (n=66) who had naloxone ordered at discharge increased from 12.7% to 57.1% | No |
| Townsel et al., 2023[18] | Labor/  Delivery/ NICU | Hospital administrator and providers | Hospital providers | - | Integrated workflow between ACS and outpatient services for pregnant persons with SUDs | ACS initiated 9 hospitalized pregnant people on buprenorphine | Yes |
| 1. **Brief interventions** | | | | | | | |
| Shenai et al., 2019[19] | Inpatient | Not specified | Pregnant patients | 31 | Brief (20-30 minute) intervention for hospitalized pregnant women with a history of substance use and trauma | Improved patient knowledge of substance use, and increased likelihood of pursing treatment | No |
| Stotts et al., 2022[20] | Labor/  Delivery/ NICU | Not specified | Hospital providers, mothers in NICU | 64 | Motivational interviewing, acceptance and commitment therapy (MIACT) intervention for mothers to facilitate SUD treatment initiation | Increased SUD treatment initiation compared to baseline (RR 1.5) | No |
| 1. **Peer support** | | | | | | | |
| Gannon et al., 2022[21] | Labor/  Delivery/ NICU | Not specified | Hospital providers and  people with OUD interacting with doulas | 23 | Doula support intervention for pregnant people with OUD | Pregnant people with OUD perceived doulas as acceptable. Pregnant people in treatment for OUD reported less stigma perceived from healthcare providers when a doula was present | No |
| Kivlighan et al., 2022[22] | Labor/  Delivery/ NICU | Hospital providers, Community organizations | Hospital providers | 75 | Trained and integrated volunteer birth companions to provide support during pregnancy/labor/delivery, including perinatal substance use education | No reported outcomes | Yes |
| Newell et al., 2022[23] | Inpatient | Hospital providers, Community organizations | Hospital providers | - | Peer support group on the hospital women’s health unit for pregnant people with SUDs | Six of 11 pregnant/previously pregnant people had increased or equal scores on the Alcoholics Anonymous Intention measure, “implying they would return to peer support groups”  3 birthing people expressed increased comfortability sharing honestly with the nursing staff... “felt accepted by  the nursing staff because of… [their] familiarity with the program” | No |
| Schulman, 2020[24] | Labor/  Delivery/ NICU | State organization (excluding PQCs), Hospital administrators, Community organizations | Hospital providers | - | Peer workforce training to provide support to pregnant people with SUDs in the community and during hospitalization, including recovery support, treatment options, and community resources | 640 certified PRCs serving one hospital and the community; 110 encounters and 50 postpartum people continue to engage with PRCs | No |
| 1. **Structural changes** | | | | | | | |
| Kroelinger et al., 2019[25] | Multiple | Federal Organizations | State administrators | 12 | Federally led learning community supporting 12 states in their development and implementation of system-level changes to OUD care | 83% of state teams developed plans to improve access to and coordination of OUD services; 75% developed plans to address health care provider awareness and training | No |
| Martin et al., 2023[26] | Inpatient | Hospital administrators, Hospital providers | Hospital providers | - | Revised hospital policy on in-hospital substance use recommending providers “respond to substance use concerns by offering patients adequate pain control, evidence-based addiction treatment, and supportive services instead of punitive responses” | No reported outcomes | Yes |
| Nichols et al., 2018[27] | Multiple | State organization | Hospital administrators and providers | - | State-level knowledge transfer collaborative to develop plans for increasing uptake of evidence-based practices related to opioid use during pregnancy | Produced tailored toolkits and best practice guidelines; provided knowledge exchange between researchers, advocates, and practitioners; reported that local coalitions and work groups implemented activities within their counties after participation | No |
| Sharp et al., 2023[28] | Labor/  Delivery/ NICU | State organizations (excluding PQCs), Hospital administrators, Hospital providers, Community organizations | Hospital administrators and providers | - | Evaluation of state implementation of CARA to provide supportive, non-punitive care coordination for pregnant people with SUDs and their families | 40% of eligible newborns with Medicaid insurance (n=2299) did not have a plan of safe care; 54% of families said someone discussed a plan of safe care with them; 26% were involved in its development | No |

**NICU**, neonatal intensive care unit; **SUDs**, substance use disorders; **OUD**, opioid use disorder; **CARA**, Comprehensive Addiction and Recovery Act; **ACS**, addiction consult service; **PRCs**, Peer Recovery Coaches; **PQC**, Perinatal Quality Collaborative

^a^ Hospital providers include physicians, nurses, social workers, care coordinators, addiction recovery specialists, certified nurse-midwives, counselors, doulas

^b^ Reported outcomes include those specific to SUD care/treatment for pregnant or birthing people. Other outcomes from the studies are not reported.

1. Ford S, Clarke L, Walsh MC, Kuhnell P, Macaluso M, Crowley M, et al. Quality Improvement Initiative to Improve Healthcare Providers' Attitudes towards Mothers with Opioid Use Disorder. Pediatr Qual Saf. 2021;6(5):e453.

2. Hostetter K, Thakkar B, Edwards C, Martin CE. Addiction curriculum design for medical students. Clin Teach. 2022;19(1):29-35.

3. Laks J, Walley AY, Bagley SM, Barber CM, Gaeta JM, Neville LA, et al. Developing a Women's Health track within addiction medicine fellowship: reflections and inspirations. Addict Sci Clin Pract. 2023;18(1):3.

4. Merritt EL, Burduli E, Purath J, Smart D. Health Care Professionals' Perceptions of Caring for Patients with Substance Use Disorders during Pregnancy. MCN Am J Matern Child Nurs. 2022;47(5):288-93.

5. Moore JD, Casanova MP, Ryu S, Smith LH, Baker RT. Examining ECHO Idaho’s perinatal substance use disorder program. Journal of Rural Mental Health. 2023;47(1):10-9.

6. Rudolf V, Plawman A, Brown L, Peterson L, Wong J, Gianutsos P, Walsh J. Improving Nursing Provider Attitudes for Pregnant Women with Opioid Use Disorder. San Francisco, CA: Substance Abuse; 2018.

7. Shuman CJ, Choberka D. Addressing Clinician Stigma Toward Perinatal Substance Use Through Art: Proof of Concept and Initial Feasibility of ArtSpective. Substance Abuse; 2022.

8. Stephenson E, Newnam K, Barker B, Brewer T. Implementing a Statewide Speakers Bureau to Address Maternal Morbidity and Mortality. Nurs Womens Health. 2022;26(3):194-204.

9. Tobin KB. Changing Neonatal Nurses' Perceptions of Caring for Infants Experiencing Neonatal Abstinence Syndrome and Their Mothers: An Evidenced-Based Practice Opportunity. Adv Neonatal Care. 2018;18(2):128-35.

10. Walsh J, Rudolf V, Sapienza D, Isner G, Vanderhoeven J, Khattar A. Improving Provider Stigma and Compassion for Substance Use in Pregnancy. Washington, DC: Substance Abuse; 2017.

11. Wands L, Pfeiffer KM, Pelkmans J. Demonstration of caring and motivational interviewing in online simulation: A cross-sectional observational study. Nurse Educ Pract. 2022;63:103370.

12. Kilaru AS, Perrone J, Kelley D, Siegel S, Lubitz SF, Mitra N, Meisel ZF. Participation in a Hospital Incentive Program for Follow-up Treatment for Opioid Use Disorder. JAMA Netw Open. 2020;3(1):e1918511.

13. Lilly CL, Ruhnke AM, Breyel J, Umer A, Leonard CE. Drug Free Moms and Babies: Qualitative and quantitative program evaluation results from a rural Appalachian state. Prev Med Rep. 2019;15:100919.

14. Naliboff JA, Tharpe N. Universal Postpartum Naloxone Provision: A Harm Reduction Quality Improvement Project. J Addict Med. 2023;17(3):360-2.

15. Paterno M, Jablonski L, Avery K, Friedmann P. Evaluation of a Nurse-Led Program for Rural Pregnant Women With Opioid Use Disorder to Improve Maternal-Neonatal Outcomes. Journal of obstetric, gynecologic, and neonatal nursing : JOGNN. 2019.

16. Snyder H, Kalmin MM, Moulin A, Campbell A, Goodman-Meza D, Padwa H, et al. Rapid Adoption of Low-Threshold Buprenorphine Treatment at California Emergency Departments Participating in the CA Bridge Program. Ann Emerg Med. 2021;78(6):759-72.

17. Stone H, Dasinger E, Brocato B, Gentle S. Initial Outcomes of a Quality Improvement Initiative to Increase Intranasal Naloxone Dispensing at Discharge among Postpartum Patients at Increased Risk of Opioid Overdose. Substance Use & Addiction Journal; 2023.

18. Townsel C, Irani S, Buis C, Lasser S, Menke N, Preston Y, et al. Partnering for the future clinic: A multidisciplinary perinatal substance use program. Gen Hosp Psychiatry. 2023;85:220-8.

19. Shenai N, Gopalan P, Glance J. Integrated Brief Intervention for PTSD and Substance Use in an Antepartum Unit. Matern Child Health J. 2019;23(5):592-6.

20. Stotts AL, Villarreal YR, Green C, Berens P, Blackwell S, Khan A, et al. Facilitating treatment initiation and reproductive care postpartum to prevent substance-exposed pregnancies: A randomized bayesian pilot trial. Drug Alcohol Depend. 2022;239:109602.

21. Gannon M, Short V, Becker M, Parikh S, McGuigan K, Hand D, et al. Doula engagement and maternal opioid use disorder (OUD): Experiences of women in OUD recovery during the perinatal period. Midwifery. 2022;106:103243.

22. Kivlighan KT, Gardner T, Murphy C, Reiss P, Griffin C, Migliaccio L. Grounded in Community: Development of a Birth Justice-Focused Volunteer Birth Companion Program. J Midwifery Womens Health. 2022;67(6):740-5.

23. Newell SM, Stem J, Lanzillotta-Rangeley J. Virtual Peer Support in Women's Health for Pregnant People and Mothers With Substance Use Disorder. Nurs Womens Health. 2022;26(3):226-33.

24. Schulman B. Improving Clinical Outcomes by Implementing NICU Peer Recovery Coaches. Journal of Obstetric, Gynecologic & Neonatal Nursing. 2020;49(6):S79-S80.

25. Kroelinger CD, Rice ME, Cox S, Hickner HR, Weber MK, Romero L, et al. State Strategies to Address Opioid Use Disorder Among Pregnant and Postpartum Women and Infants Prenatally Exposed to Substances, Including Infants with Neonatal Abstinence Syndrome. MMWR Morb Mortal Wkly Rep. 2019;68(36):777-83.

26. Martin M, Snyder HR, Otway G, Holpit L, Day LW, Seidman D. In-hospital Substance Use Policies: An Opportunity to Advance Equity, Reduce Stigma, and Offer Evidence-based Addiction Care. J Addict Med. 2023;17(1):10-2.

27. Nichols T, Gringle M, Harvey G, Marshall A. North Carolina’s response to maternal opioid use: Mapping a KT intervention. Washington, DC: Implementation Science; 2018.

28. Sharp N, Fuchs J, Drake A. An Implementation Evaluation of the Comprehensive Addiction Recovery Act (CARA) Policy in New Mexico. Matern Child Health J. 2023;27(Suppl 1):113-21.
