# Supplementary material for: Implementing substance use services into acute care settings for pregnant and birthing people: A systematic scoping review of implementation and quality improvement strategies
Source: PLoS One. 2026 Mar 13;21(3):e0344389. doi: 10.1371/journal.pone.0344389 (PMC12987426; doi:10.1371/journal.pone.0344389)
Supplement: Attachment — Submitted filename: Response to Reviewers.docx [file pone.0344389.s006.docx]

**RESPONSE TO REVIEWERS**

**Editor's comments**

1. Data availability statement
Authors are required to make all data underlying the findings described fully available, without restriction, and from the time of publication. PLOS allows rare exceptions to address legal and ethical concerns. See the PLOS Data Policy and FAQ for detailed information. Kindly follow the instructions on data availability statement and provide clear reasons as to why the data cannot be made available.

We’ve added a data availability statement on page 37 (line 955) stating “All data analyzed in this review are from previously published studies, which are cited throughout the manuscript. No new data were generated.”

2. Please ensure consistency of the subject, research objective, methods, and conclusions made when revising. Your objective does mention that you are looking at strategies to "promote uptake of equitable, evidence-informed care for pregnant or birth people with SUDS in acute hospital-based settings". From this objective we expect the focus of the paper to be on strategies to increase demand and hence uptake. Your response was that you "summarized the outcomes that were measured by the studies (i.e., changes to provider knowledge or attitudes, uptake of medications for SUD treatment)." This is not clear since your objective has specified the outcome to be "adoption/uptake". For every strategy listed, the readers need to see how they are related to adoption/uptake of equitable, evidence-informed care for pregnant or birth people with SUDS. Even though scoping reviews cover wide topics, you are still limited to what your objectives are. Kindly revise accordingly.

Thank you for this comment. It was very helpful in guiding how we clarified and aligned our objective, outcomes, and overall framing. In response, we have edited relevant sections throughout the manuscript to ensure consistency across subject, objective, methods, results and conclusions.

We clarified that the subject of our review is the integration of SUD services (i.e., medications, referrals to treatment, and overdose prevention) into acute care settings for pregnant and birthing people (page 4). We also updated the manuscript’s title to more clearly articulate this: *“Integrating substance use disorder services into acute care settings for pregnant and birthing people: A systematic scoping review of implementation and quality improvement strategies”*

Previously, our use of the term “uptake/demand” in the objective was ambiguous and did not fully reflect the focus of our analysis. We’ve more clearly stated our objective on page 5:

“To address this gap, we used scoping methods to systematically review studies that used an implementation or QI strategy to **integrate SUD services for pregnant or birthing people into an acute care setting.** Scoping methods were chosen to review a body of research that is in its early stage of development, and to identify gaps in measurement and outcomes that would aid future research and implementation. [55] We aimed to 1) characterize and describe the implemented strategies; 2) assess the inclusion of racial equity in study design and implementation; 3) summarize measures and outcomes used to evaluate implementation; and 4) identify reported barriers and facilitators to implementation, sustainability, and/or scale-up.”

To align with this objective, we’ve also more explicitly specified our outcomes on page 8:

“Our outcomes of interest were 1) implementation-related; and 2) the extent to which a racial and ethnic equity lens was incorporated into the study design and implementation. We extracted any reported implementation outcomes that related to SUD services for pregnant and birthing people, including acceptability, feasibility, adoption, and sustainability (see S2 File). [65] We also noted the level at which the outcome was measured, when applicable (i.e., patient-, provider-, hospital-, or community-level). To assess how studies acknowledged and addressed racial and ethnic equity in their design and implementation, we extracted explicit or implicit mentions of racial equity using Shelton et al.’s recommendations for addressing racism in implementation science as a guide…”

We’ve also revised Table 2 to include columns with both outcomes of interest (i.e., implementation-related outcomes and racial equity in design/implementation).

We thank you again for this comment and hope these changes more clearly align the manuscript with our stated objective and research focus.

**Reviewer #1**

Thanks for your responses and changes in response to previous comments.

**1. Introduction**
Thanks for the clarifications as outlined in your response letter. I appreciated the attempt to specify which recommendations were included as follows "... including SUD treatment referrals, medications for addiction treatment, harm reduction resources and education, and non-punitive/non-stigmatizing care, could help improve quality of care and address inequitable SUD treatment gaps. [11, 12, 30]". These are broad categories in themselves and overlapping. For example, medications to address SUD are generally considered as harm reduction strategies. I would suggest picking 2 or 3 specific recommendations such as medications or referrals to addiction treatment programs and then evaluating how these were implemented.

Thank you again for your review and helpful comments. Based on your suggestion, we’ve narrowed our focus to specific clinical practices: medications, referrals to treatment, and overdose prevention. Through the manuscript, we refer to these as “SUD services”. While we recognize this still covers a broad range of SUD practices, we feel that these are often overlapping and implemented together. We hope that by limiting our review to these 3 key elements, we have more clearly evaluated how they were implemented.

You stated that "We exclude aspects of care that are best practices for pregnant and birthing people with SUDs, but that do not directly address SUDs"; however, in the section on system level changes, the use of supports during labour and delivery to help with intrapartum ?pain was included which is not directly related to SUD.

Thanks for this comment. Perhaps we created confusion because we didn’t define “plans of safe care” or provide enough details about the hospital policy by Martin et al. described in this section. In response, we’ve added a line describing the purpose of plans of safe care:

“Another study evaluated the state’s implementation of the Comprehensive Addiction Recovery Act (CARA) and Plans of Safe Care which are intended to provide supportive, non-punitive SUD treatment referrals and care coordination for pregnant people with SUDs and their families” Page 21, Line 406-409.

We’ve also added more information about the hospital policy study we included:

“The revised hospital policy recommended hospital providers respond to patients, including those who are pregnant and racialized, who were using substances during hospitalization by offering pain and withdrawal support, addiction treatments, and supportive services for the SUD.” Page 21, Line 398-401.

We hope these changes address your comment.

**2. Methodology**
You have indicated that you followed the PRISM flowchart for scoping reviews; however, the checklist for this methodology still requires clear indication of "Data items in section 11 - List and define all variables for which data were sought and any assumptions and simplifications made." Therefore, the outcomes of interest for this review would be helpful.

Thank you for bringing this to our attention. We’ve added our outcomes of interest to the data extraction section on page 8 (these are also listed in Supplementary File 2):

“Our outcomes of interest were 1) implementation-related; and 2) the extent to which a racial and ethnic equity lens was incorporated into the study design and implementation. We extracted any reported implementation outcomes that related to SUD services for pregnant and birthing people, including acceptability, feasibility, adoption, and sustainability (see S2 File). [65] We also noted the level at which the outcome was measured, when applicable (i.e., patient-, provider-, hospital-, or community-level). To assess how studies acknowledged and addressed racial and ethnic equity in their design and implementation, we extracted explicit or implicit mentions of racial equity using Shelton et al.’s recommendations for addressing racism in implementation science as a guide.

We also updated the PRISMA-ScR checklist to reflect the correct page numbers.

Overall, organizing articles into 3 categories provided a structure for this review but the 3 topics themselves are very broad. Changing provider attitudes was not linked to change in practice which would be more meaningful in terms of evidence for educational interventions or persistent change in attitudes at months post-intervention. These specifics would be more meaningful in terms of determining what has been effective and what should be changed in future interventions.

As mentioned previously, this review's broad focus leads to inclusion of studies with too much heterogeneity in terms of audience for interventions and types of interventions to make meaningful summary statements.

Thank you again for this comment – it has significantly helped improve the clarity and organization of our manuscript. After carefully reflecting on your comments, we reorganized our results to better align with the objectives of the paper (i.e., characterizing strategies used to integrate SUD services). The results are now organized into 5 groups reflecting the type of strategy that was implemented: 1) education and learning collaboratives; 2) clinical pathways or workflows; 3) brief interventions; 4) peer support; and 5) structural changes. To guide this reclassification, we employed the Expert Recommendations for Implementing Change framework (referenced on page 9).

We hope this reorganization will help readers better understand the types of strategies used, the outcomes that were and were not measured, and where next steps are needed to assess effectiveness, adoption etc. We recognize that these strategies themselves can be applied to multiple aspects of SUD service delivery and intentionally include a range to illustrate what has been implemented and what gaps remain. Given this is a scoping review, we hope this broadly covers the extent of variability so that more targeted, bundled interventions can be studied or implemented.

We also revised the discussion to clarify the intended use of this review as a foundational step toward more targeted implementation science and quality improvement efforts, and added to our limitations section on page 30, lines 631-634 to more directly address study heterogeneity:

“Finally, one key limitation is the heterogeneity of the included studies. While this was an intentional aspect of our scoping methodology, it limits our ability to draw conclusions about the effectiveness of specific strategies. Future systematic or realist reviews may be better suited to evaluate the effectiveness and implementation of specific interventions in more detail.”

We hope our changes help clarify the utility and limitations of this review.

**Reviewer #2**

 I thank the authors for thoroughly addressing reviewer comments and editing the manuscript thoughtfully.

We thank you again for your review.
