# Supplementary material for: Implementing substance use services into acute care settings for pregnant and birthing people: A systematic scoping review of implementation and quality improvement strategies
Source: PLoS One. 2026 Mar 13;21(3):e0344389. doi: 10.1371/journal.pone.0344389 (PMC12987426; doi:10.1371/journal.pone.0344389)
Supplement: Attachment — Submitted filename: Response_to_Reviewers_auresp_3.docx [file pone.0344389.s007.docx]

**RESPONSE TO REVIEWERS**

**Reviewer #1**

**Thank you to the authors for addressing the reviewer comments and making the indicated revisions to this manuscript.**

**Overall, the clarifying points made this manuscript easier to follow. However, there are a couple of brief feedback points that I would like to highlight.**

1. **Title: thanks for revising the title; however, I am not sure if "Integrating" is the correct descriptive word of the strategies, the description of the types of services lead to perhaps "implementing" or "adopting" since some of the strategies occurred only once (eg. educational initiatives).**

Thank you again for your review and suggestions. We have replaced “integrating” with “implementing” in the title and throughout the manuscript.

2. **Introduction: The intro section is very long - 3 page and there are many facts presented; however, it would be easier to follow where the article is going by having a more cohesive presentation.
   The terminology "acute care settings" sounds confusing since the main focus is hospital inpatient care so why not label it as such. Acute care settings refer to both outpatient and inpatient settings, but this review is aimed at inpatient hospital setting primarily.**

Thank you for this feedback. We have made edits to the content and structure of the introduction, while maintaining a previous reviewer’s suggestion to add more depth. We have also defined “acute care settings” earlier in the introduction, specifying that acute care in our study refers to inpatient, labor/delivery, or emergency care only. Although there were only a few studies that focused on the emergency department, we felt that specifying hospital inpatient care did not accurately describe those studies, and so opted to keep “acute care”. We hope that by defining it earlier in the introduction, we have made this less confusing.

3. **Outcomes under data extraction are mentioned very vaguely. It appears that there were no predefined outcomes and that researchers accepted all published outcomes of various strategies.**

Thanks for this comment. You are correct that we did not pre-define specific outcomes. Because we used scoping methodology, we examined all implementation outcomes, as defined by Proctor et al. 2011, in the included studies. We have added clarity in this section (lines 179-183) by referencing the framework we used to define implementation outcomes:

“We extracted reported implementation outcomes, as defined by Proctor et al., that related to implementation of substance use services for pregnant and birthing people, including acceptability, feasibility, adoption, and sustainability (see S2 File). [52] We also noted the level at which the outcome was measured, when applicable (i.e., patient-, provider-, hospital-, or community-level).

4. **Grouping studies into five types makes the vast amount of information provided much easier to follow and to understand. Overall, there is a heterogeneous mix of services and strategies presented which suggests a wide variation in types of strategies even within one category which lends to difficulty in future studies.**

Thanks for this comment. We agree – there is a heterogeneous mix of services and strategies presented. While this is noted as a limitation of our study (lines 630-634), we also believe this is an important finding.

To this end, we have made recommendations for future studies throughout our discussion and conclusion sections. For example, in our discussion, we emphasized that there are many heterogenous interventions that focus on shifting provider attitudes and knowledge, however, few have successfully evaluated which of these strategies led to sustained adoption or improved patient outcomes, and so more rigorous studies are needed to determine how to best use these strategies (lines 529-534; 551-558; 570-574). We also noted that there was wide variability in how implementation outcomes were defined and measured within and across strategies limiting comparisons. In response, we have provided recommendations for future research on lines 607-614:

*Strategies and implementation outcomes were often poorly defined and heterogeneous, making it difficult to compare across studies or to determine mechanisms that made a strategy successful within a specific context. Using existing implementation frameworks for specifying strategies and outcomes (e.g., Taxonomy of Implementation Outcomes [51], RE-AIM [95]) could help standardize language and measurement, and allow for more systematic comparisons between studies. [96] Collaboration between implementation science, QI researchers, and healthcare administrators could help address this gap. [34, 97]*

Overall, we believe our study identifies a clear gap in the current literature and recommend that future research focuses on more clearly defined interventions and outcomes so that categories of strategies can be more rigorously evaluated and compared (lines 643-647). We hope this has addressed your concerns regarding the heterogeneity of strategies. Thank you again for your thoughtful and thorough feedback that has improved our manuscript.

**Reviewer #2**

**(No Response)**
